# Comparing Historical and Contemporary Observations of Avian Fauna on the Yáláƛi (Goose Island) Archipelago, British Columbia, Canada

**DOI:** 10.1002/ece3.70464

**Published:** 2024-12-04

**Authors:** Debora S. Obrist, Elizabeth Jane Pendray, Rachel D. Field, William Housty, Allison M. Dennert, Gerald W. Scoville, Chris T. Darimont, John D. Reynolds

**Affiliations:** ^1^ Earth to Ocean Research Group, Department of Biological Sciences Simon Fraser University Burnaby British Columbia Canada; ^2^ Department of Evolutionary Biology and Environmental Studies University of Zürich Zürich Switzerland; ^3^ Pacific Salmon Foundation Vancouver British Columbia Canada; ^4^ The Okanagan Institute for Biodiversity, Resilience and Ecosystem Services, Irving K. Barber School of Science University of British Columbia Okanagan British Columbia Canada; ^5^ Department of Biology University of British Columbia Okanagan Kelowna British Columbia Canada; ^6^ Heiltsuk Integrated Resource Management Department Bella Bella British Columbia Canada; ^7^ Raincoast Conservation Foundation Sidney British Columbia Canada; ^8^ Department of Biological Sciences Central Washington University Ellensburg Washington USA; ^9^ Department of Geography University of Victoria Victoria British Columbia Canada

**Keywords:** avian ecology, global change, historical ecological data, Indigenous knowledge, museum collections, point count surveys

## Abstract

In an era of global change, historical natural history data can improve our understanding of ecological phenomena, particularly when evaluated with contemporary Indigenous and place‐based knowledge. The Yáláƛi (Goose Island) Archipelago is a group of islands in Heiltsuk (Haíɫzaqv) territory on the Central Coast of British Columbia, Canada. Not only has this region been important to the Heiltsuk for millennia but also it is both a federally and internationally recognized important bird area. In this study, we compare data collected by Charles J. Guiguet, a biologist who documented bird communities at Yáláƛi in the summer of 1948, to three different contemporary surveys and to citizen‐science data. We find that the relative abundances of forest bird species (i.e., birds that use the terrestrial island ecosystems) in 1948 differed to those observed in systematic surveys in 2011. While Orange‐crowned Warblers, Dark‐eyed Juncos, and Red Crossbills comprised 55% of detections by Guiguet in 1948, the three most abundant species in 2011 were Bald Eagles, Varied Thrushes, and Pacific Wrens, and these accounted for only 25% of detections. Although we could not make a quantitative comparison, we provide summaries of each species observed in surveys or reported on eBird. We also incorporate Heiltsuk place‐based knowledge to enrich our discussion of the variability in bird communities over time, from how changes in mammal communities and human use may have shaped vegetation dynamics to how large‐scale natural phenomena impacted topography. To understand which birds are present and how their communities are changing over time, we recommend continued monitoring of the bird communities at Yáláƛi.

## Graphical abstract

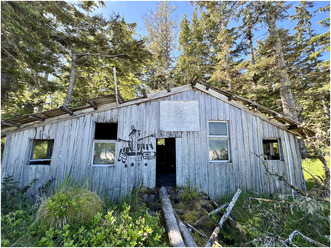

## 1 Introduction

Long‐term monitoring and use of historical datasets facilitate a deeper understanding of the drivers and effects of global change on biodiversity in many ecosystems, at multiple scales, and along various axes of biodiversity (Magurran et al. 2010). For instance, comparing kelp surveys from 1993 to 1995 to those from 2017 to 2018, Starko et al. (2019) found that broad‐scale stressors resulted in a decrease in kelp diversity in Barkley Sound, British Columbia (BC), Canada. Similarly, Harley (2011) found significant climate change‐driven shifts in vertical zonation of intertidal invertebrate communities by comparing historical (1957–1958) to contemporary (2009–2010) datasets. However, historical datasets do not only consist of systematic species abundance surveys. Price et al. (2021), for example, used century‐old fish scales to track population and life‐history diversity of Pacific salmon in the Skeena River watershed, BC, and found a contemporary decrease in both. The Mountain Legacy Project, a photographic collection of western Canadian mountains since 1888, has facilitated studies such as Trant, Higgs, and Starzomski (2020) and Fortin (2018), who used these data to track changes in montane ecosystem diversity and predict songbird occurrences over time, respectively. Here, we showcase how natural history observations in different periods can complement systematic surveys, yielding insight into ecosystem change.

Long before natural history and science were conceived by western societies, place‐based practices of Indigenous peoples included many of the same processes, like observation, measurement, and experimentation (Agrawal 1995). Given long‐term resource use and occupation of landscapes and seascapes, as well as vibrant, contemporary practice, Indigenous Peoples have accumulated and continue to refine their Indigenous knowledge (Thompson et al. 2019). Accordingly, our understanding of how ecosystems change over time becomes much more comprehensive with a “Two‐Eyed Seeing” approach that centers the inclusion of place‐based knowledge, including Indigenous knowledge and epistemology (Bartlett, Marshall, and Marshall 2012; Reid et al. 2021). For instance, through a combination of local ecological knowledge, traditional ecological knowledge, and non‐Indigenous (“western”) science, Service et al. (2014) found evidence for grizzly bear (*Ursus arctos horribilis*) range expansion on islands in coastal BC, stating that the incorporation of several forms of knowledge provides higher spatial and temporal resolution than any singular knowledge system on its own. Indigenous Peoples in coastal BC have been stewards of the land since time immemorial (Gauvreau et al. 2023). This extensive timeframe underscores millennia of direct experience engaging with biophysical and ecological processes, landscapes, ecosystems, and species (Atleo 2011; Jessen, Ban, et al. 2022), making Indigenous Peoples very attuned to ecological change (Turner and Clifton 2009). Often, western science—described as the “curious little sister” of Indigenous science—merely substantiates what Indigenous Peoples have long understood, through centuries of collective knowledge and shared learning (Housty 2021). Indeed, in Northern British Columbia and Southern Alaska, Indigenous community residents have detected significant ecological changes over their lifetimes, including in the behaviors, distributions, and availability of important plants and animals (Wyllie De Echeverria and Thornton 2019). Given such detailed insight, combining the use of historical datasets with Indigenous knowledge provides considerable value in addressing many ecological questions, particularly in the face of an assortment of global stressors and resulting changes (Gadgil, Berkes, and Folke 1993; Ban et al. 2018; Ogar, Pecl, and Mustonen 2020; Petzold et al. 2020; Jessen, Ban, et al. 2022; Jessen, Service, et al. 2022).

Goose Island is the largest island of Yáláƛi, the Goose Island Archipelago, a group of islands in Heiltsuk (Haíɫzaqv) First Nation territory on the Central Coast of BC, Canada. As an Important Bird Area (Important Bird Areas Canada n.d.), Yáláƛi is an important cultural and migratory hotspot for both the Heiltsuk and for birds, who have been using the islands seasonally for millennia (Heiltsuk Traditional Use Study, Heiltsuk Nation 1998‐present). Until the 1920s–1940s, the entire nearby community of Wágļísļa (Bella Bella), including author W. Housty's late great‐grandfather, would pack up and travel to Yáláƛi for 6–8 weeks every summer, bringing with them school, church, and play. Over time, the Heiltsuk people have observed that this area often experiences changes in kelp beds and mollusks, among other things, prior to elsewhere in the territory (Heiltsuk Traditional Use Study, Heiltsuk Nation 1998‐present). It is therefore possible that changes in bird communities may be observed here first too. As such, part of the impetus for this project was to determine if Yáláƛi could serve as a sentinel location for change across Heiltsuk territory.

In the summer of 1948, Charles J. Guiguet (1915–1999, Figure 1), a naturalist and taxidermist of European descent, spent 4 months at Yáláƛi, collecting data on behalf of BC's Provincial Museum of Natural History and Anthropology (now the Royal BC Museum). Although not by invitation or permission of the Heiltsuk, he surveyed plants, mammals, and birds, and published his observations in his Master's thesis and a collection of field books. Although Guiguet's historical dataset provides only a snapshot in time, by combining his observations with contemporary surveys and community science data, and by interpreting the results through a lens that focuses on local knowledge, we can gain insights into how ecological communities of birds on the Goose Islands might have changed in at least the last 75 years.

**FIGURE 1:**
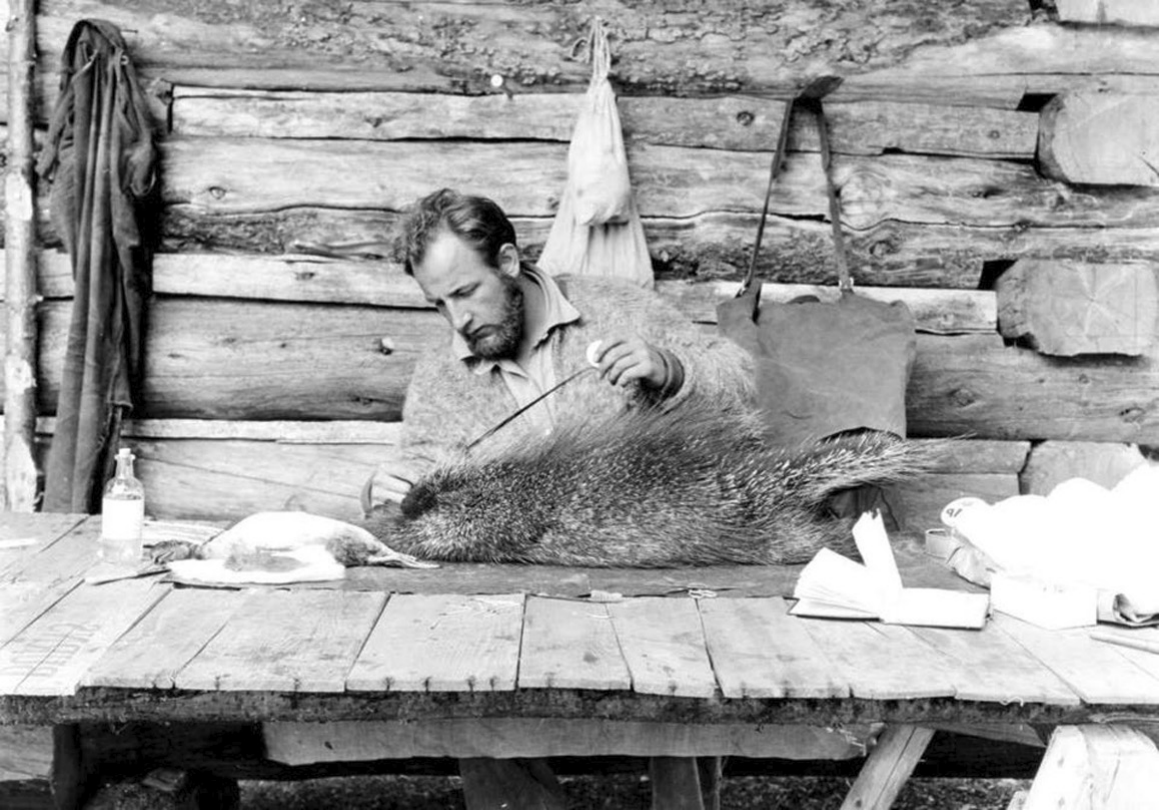
Charles Guiguet (1916–1999) in Tweedsmuir Provincial Park, near Stuie, British Columbia. 16 September 1938. B.C. Archives, Province of British Columbia, photo number 6‐03674. Copyright: Public Domain.

This project was initially conceived in response to a proposed natural gas condensate and diluted bitumen pipeline project in the early 2010s. The Heiltsuk Nation was deeply concerned about the potential impacts to ecosystems should an oil spill occur because of increased tanker ship traffic. This pipeline project was never built. However, a catastrophic diesel spill in nearby Seaforth Channel at Q̓vúqvái (Gale Creek) in 2016—as well as a subsequent near‐miss in 2017 when a loaded fuel barge broke free off Gosling Rocks during a storm—have highlighted the importance of collecting baseline biodiversity data for adequately measuring potential impacts of anthropogenic environmental disasters. As such, our study was guided by three main objectives. First, we wanted to consolidate baseline biodiversity data about birds. Second, we wanted to evaluate the potential to detect changes in bird communities over time at Yáláƛi. Finally, we sought to provide recommendations, in consultation with the Heiltsuk Integrated Resource Management Department (HIRMD) for continued monitoring of bird populations in a way that aligned with Heiltsuk needs and values.

To achieve our goals, here we compile and compare detections from historical avian surveys performed by Guiguet in 1948 with those from three contemporary surveys—one in 2010, by a Heiltsuk‐led monitoring and stewardship initiative called CoastWatch (led by W. Housty and G. Scoville), and two by Simon Fraser University (SFU) researchers in 2011 (R. Field and J. Reynolds) and 2015 (J. Reynolds)—as well as with modern community science observations from eBird (2010–2023). We interpret the results in consideration of local knowledge, including a series of surveys known as the traditional use studies conducted with community elders in the 1990s, now maintained by HIRMD (Heiltsuk Traditional Use Study, Heiltsuk Nation 1998‐present). Since Guiguet conducted systematic surveys only for forest birds, the 2011 Simon Fraser University surveys were designed to replicate these in the same habitat types. We present that comparison here. Specifically, we compare the relative proportions of terrestrial species detected in 1948 and 2011. Additionally, we use records from all available surveys at Yáláƛi to evaluate the presence/absence of all avian species, including seabirds, across the 75‐year period between Guiguet's surveys and the most recent eBird observations. We discuss several possible reasons for changes in bird communities throughout this time period, including changes in mammal communities, which were documented by both Guiguet in 1948 and by CoastWatch in 2010. We also address some of the challenges associated with comparisons of historical and contemporary data. Finally, we make some specific recommendations for future monitoring efforts of avian species at Yáláƛi.

## 2 Positionality

We are a team of ecologists and environmental professionals connected in our dedication to the conservation of British Columbia's coastal ecosystems, including those in Heiltsuk First Nation territory. Our research team consists primarily of non‐Indigenous members: D. Obrist, E. Pendray, R. Field, A. Dennert, G. Scoville, C. Darimont, and J. Reynolds, residing on the territories of the šxʷməθkʷəy̓əmaɁɬ təməxʷ (Musqueam), Sḵwx̱wú7mesh (Squamish), səlilwətaɬ (Tsleil‐Waututh), Lək̓ʷəŋən (Songhees and Esquimalt), W̱SÁNEĆ (Saanich), Tk'emlúps te Secwe̓pemc, Nlaka'pamux, and Yakama Nations. W. Housty, a member of the Heiltsuk First Nation, resides in Wágļísļa (Bella Bella), BC, and works as Associate Director of the Heiltsuk Integrated Resource Management Department (HIRMD). W. Housty connected the author team with sources of local and Indigenous knowledge about the birds of Yáláƛi, including the contribution of personal observations, which are explicitly noted throughout this manuscript. Other knowledge we have included in this manuscript comes from both Heiltsuk and non‐Heiltsuk colleagues and community members who have connections and personal experiences interacting with the land and seas of Yáláƛi, and is marked by a personal communication citation. Knowledge holders who contributed to this work and to the Heiltsuk Traditional Use Studies all had many years of lived experience in Haílzaqv territory, including at Yáláƛi. Activities at Yáláƛi commonly included fishing, hunting, intertidal harvesting, medicinal plant gathering, and more, and were frequently conducted in a multigenerational learning context. All those who have directly provided knowledge have also given us permission to use it in this manuscript.

This project was initiated in response to a proposed natural gas condensate and diluted bitumen pipeline in the early 2010s and was driven by concerns about potential ecosystem impacts from increased tanker traffic and oil spills. W. Housty and G. Scoville surveyed birds (and many other taxa) at Yáláƛi on behalf of CoastWatch, a Heiltsuk scientific research initiative that recorded ecological data with the intent of guiding stewardship on both land and sea. By offering essential knowledge and skills, this non‐profit society empowered members of the Heiltsuk Nation to actively engage in science, resource management, and conservation planning in their homeland, thereby also illustrating practices that underlie vibrant, contemporary place‐based knowledge.

L. Jorgensen, W. Housty's father and long‐term resident of Wágļísļa, originally conceptualized a comparison of historical and contemporary bird communities using the Guiguet dataset. L. Jorgensen connected J. Reynolds, a non‐Indigenous professor with a long history of research collaborations with HIRMD, with CoastWatch, to engage in this work. Although the initial proposed pipeline was not built, this project evolved into an effort to consolidate baseline biodiversity data, consider how Yáláƛi could serve as a sentinel location in the context of climate change in Heiltsuk territory, and ultimately develop bird monitoring recommendations that align with the broader resource management goals of the Heiltsuk Nation.

## 3 Methods

### 3.1 Study Site

**FIGURE 2:**
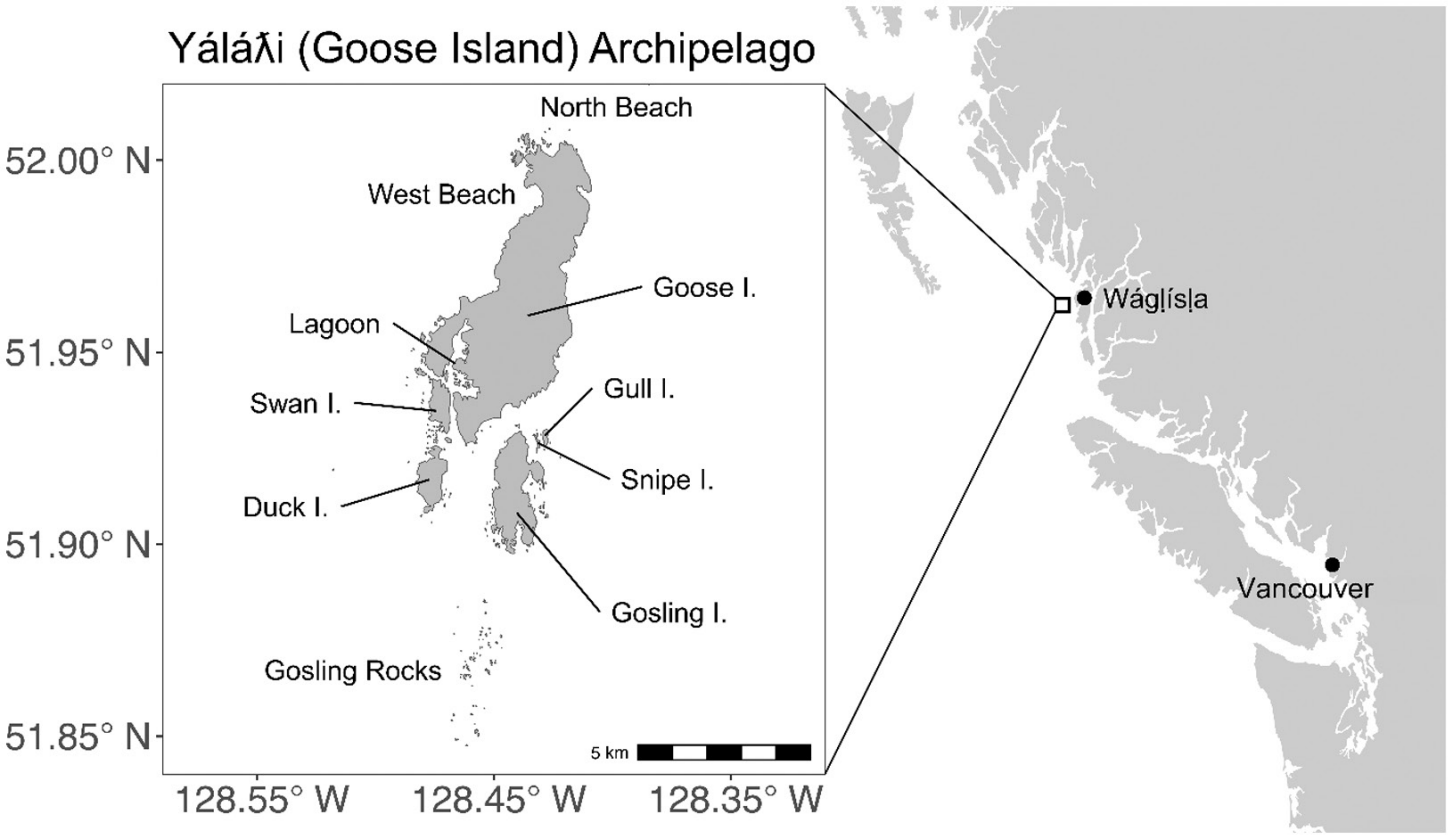
Yáláƛi (Goose Island) Archipelago on the central coast of British Columbia, Canada.

Yáláƛi (ya‐LA‐tclee, sometimes anglicized as “yellertlee,” 51.621° N, 128.825° W, Figure 2) is an archipelago in Heiltsuk (Haíɫzaqv) First Nation territory, about 30 km southwest of the town of Wágļísļa (Bella Bella), British Columbia (BC). For the pronunciation of Yáláƛi and Wágļísļa from First Voices, respectively, visit the following links: https://www.firstvoices.com/hailzaqvla/words/dcc2936c‐6bab‐4bff‐b2ac‐89fa1bfc11a0 and https://www.firstvoices.com/hailzaqvla/words/38ad4314‐04e2‐49ad‐8072‐746ee3c13404. Also known as the Goose Islands, this archipelago consists of the western‐most group of islands at this latitude; thus, to the west, it is exposed to the open sea. To the east, Yáláƛi is isolated from the first substantial landmass (Hunter Island, roughly 13 km away) by Queen's Sound and is roughly 38 km from the mainland. The archipelago consists of six main islands: Goose Island (18.3 km^2^), Gosling Island (3.1 km^2^), Duck Island (1.1 km^2^), Swan Island (0.7 km^2^), Gull Island (0.07 km^2^), and Snipe Island (0.03 km^2^), as well as many smaller islands and islets. Except for Duck Island, these islands are all connected at low tide (Guiguet 1953) and are predominantly surrounded by rocky shorelines. One important area is known as the “lagoon” on Goose Island, a large tidal mud and sand flat in the southwestern portion of the island. The Gosling Rocks are a series of rocky reefs and islets south of the main island group, extending across a few kilometers. Finally, the Goose Island Banks refer to an offshore shelf that lies approximately 20–50 km to the southwest of the main group of islands. For the purposes of this project, we do not consider birds on the Banks, although Guiguet made some offshore trips and observations in that direction.

Yáláƛi has a long history of human habitation and use (Heiltsuk Traditional Use Study, Heiltsuk Nation 1998‐present). The Heiltsuk have lived on and used these islands and their resources since time immemorial. Several islands have hosted large villages in the past. Since the forced relocation of the Heiltsuk by the Canadian federal government to Wágļísļa (Bella Bella) in the late 19th and early 20th centuries, only a few buildings remain, with intermittent use. These include one cabin in the lagoon and another at the north beach of the island used for subsistence and rediscovery camps. There has only been Heiltsuk traditional use of trees, including cutting select trees for building canoes and longhouses, bark‐stripping for weaving and medicinal purposes, with no industrial logging activity ever taking place (Heiltsuk Traditional Use Study, Heiltsuk Nation 1998‐present). The remote archipelago is also visited occasionally by kayakers.

**FIGURE 3:**
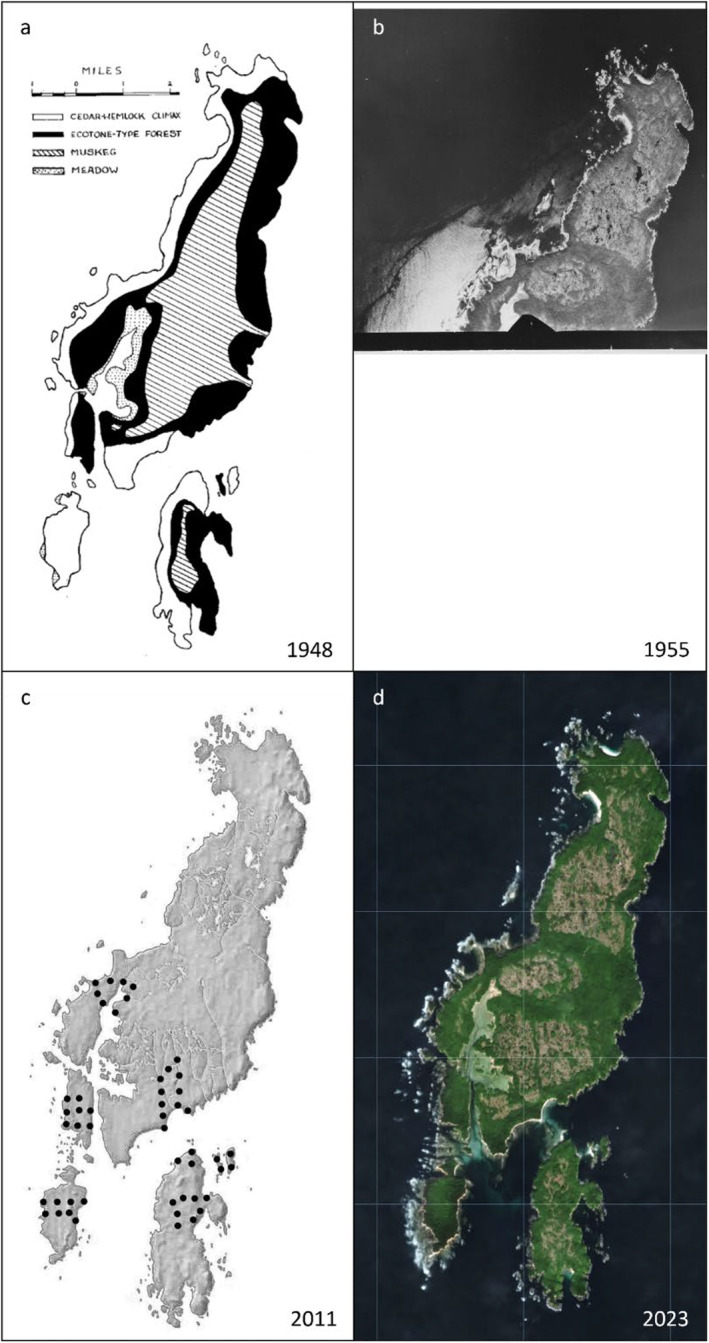
Dominant vegetation communities at Yáláƛi (Goose Island Archipelago), (a) as drawn by C.J. Guiguet in 1948 (Guiguet 1953), (b) as captured in a 1955 aerial image of northern Goose Island, obtained from the National Air Photo Library of Natural Resources Canada (reproduced with permission of the Department of Natural Resources Canada 2024), (c) an overlay showing the point count locations surveyed in authors J. Reynolds and R. Field's 2011 fieldwork with Simon Fraser University, and (d) as observed from the Copernicus satellite, Harmonized Sentinel‐2 MSI: MultiSpectral Instrument, Level‐2A in 2023. In (a), the vegetation types are depicted as follows: Cedar hemlock forest is white, ecotone forest is black, muskeg (bog) is cross hatched, and meadow is stippled.

**FIGURE A1:**
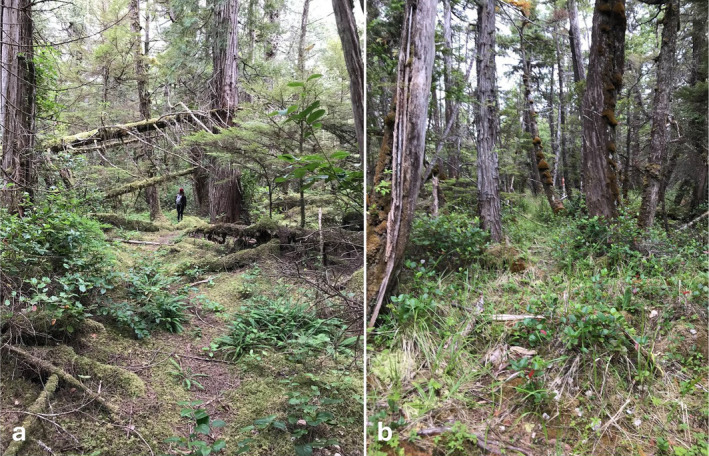
Climax forest (a) vs. ecotone forest (b) at Goose Island, Yáláƛi. Climax forest at Yáláƛi is dominated by Sitka Spruce (*Picea sitchensis*), Western Hemlock (*Tsuga heterophylla*), and Western Red‐cedar (*Thuja plicata*), while the ecotone forest additionally hosts Yellow Cedar (*Chamaecyparis nootkatensis*) and Shore Pine (*Pinus contorta*). Photos by A. Dennert (August 2018).

**FIGURE A2:**
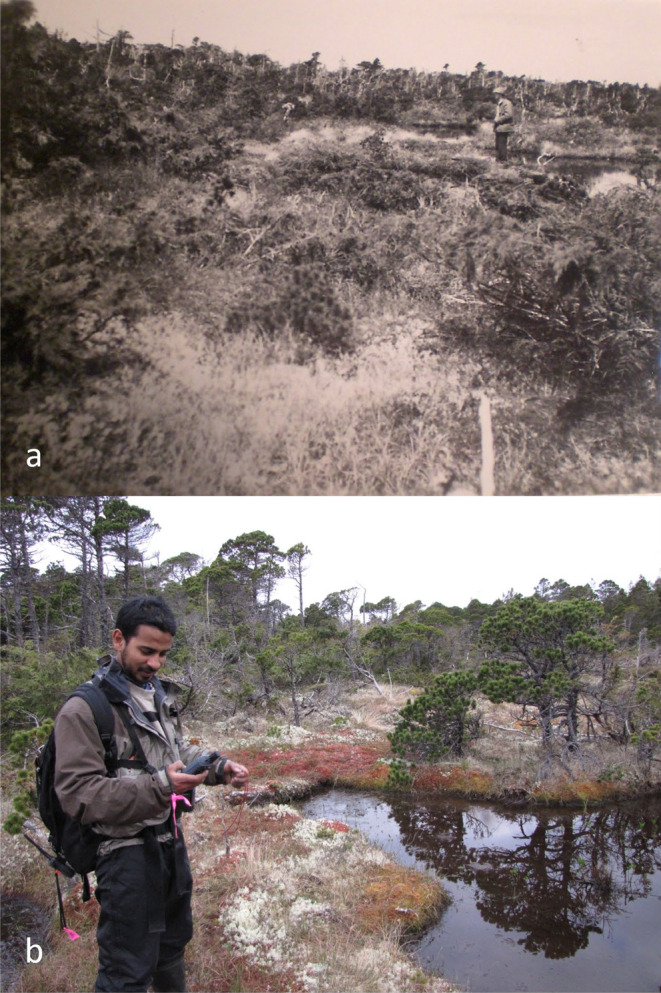
Field ecologists in the Goose Island bog in (a) 1948 and (b) 2011 (Ummat Somjee, photo by J. Reynolds.)

This island group is part of the hypermaritime subzone of the Coastal Western Hemlock biogeoclimatic zone (Banner et al. 1993). The moderating influence of the Pacific Ocean means that winters are mild, summers are cool, and over 3 m of rainfall occurs annually (Pojar, Klinka, and Meidinger 1987). Although Duck Island has some of the largest trees in all of Haíɫzaqv territory, the trees on Goose and Gosling Island are gnarled and scrubby in comparison (W. Housty, personal observation). An approximately 25‐ to 300‐m fringe of coniferous climax forest dominated by Sitka Spruce (*Picea sitchensis*), Western Hemlock (*Tsuga heterophylla*), and Western Red‐cedar (*Thuja plicata*) expands down the west coasts of Goose and Gosling Island (Figure 3a). Next, a layer of what Guiguet termed ecotone‐type forest surrounds an extensive bog habitat at the islands' centers (called a “muskeg” by Guiguet). The ecotone forest additionally hosts Yellow Cedar (*Chamaecyparis nootkatensis*) and Shore Pine (*Pinus contorta*) (Figure A1). The bogs host typical bog species including Bog‐laurel (*Kalmia microphylla*), Tufted Clubrush (*Trichophorum cespitosum*), Common Cotton‐grass (*Eriophorum angustifolium*), and various species of *Sphagnum* mosses (see Figure A2 for Yáláƛi bog photos from 1948 and 2011).

### 3.2 Bird Surveys

#### 3.2.1 1948 Surveys Led by Charles J. Guiguet

Between May 13th and the end of August 1948, C.J. Guiguet and his assistant, P. Martin, conducted extensive surveys of many taxa at Yáláƛi. This included the collection of many specimens, most of which are now housed at the Royal BC Museum in Victoria, BC. The Yáláƛi mammal communities were the primary focus of Guiguet's first publication from the area (Guiguet 1950, summarized in Table A1). However, Guiguet also surveyed birds, fish, and invertebrates. The methods, which we summarize here, and results of his bird surveys, which we use as data points, are published in Guiguet (1953). He wanted to determine the relative numbers of the different species in each of the main habitat types on Goose Island. From May 11 to June 11, 1948, Guiguet and Martin recorded the number, kind, and habitat of various birds encountered throughout their mammal and vegetation surveys. Starting June 11th, Guiguet decided that it made sense to incorporate a more systematic approach and selected a series of plots, at random, in the four major habitats (i.e., the coniferous forest, the forest‐muskeg ecotone, the muskeg, and the meadow). For the other habitats (i.e., rocky seashore, tidal mud and sand flats, and boulder beach), Guiguet noted that these were primarily used by migratory shorebirds. As such, he kept records, but did not conduct systematic surveys in these habitats.

Guiguet and Martin were well‐trained ornithologists and identified birds by both sight and sound throughout Yáláƛi. In conifer forests, Guiguet designated five point count stations within randomly distributed plots that were 150 yards (137 m) long and 50 yards (46 m) wide. Guiguet and Martin conducted all point counts together, by remaining silent for 1 min, and recording all birds seen and heard. They then mimicked northern pygmy‐owl calls for an additional 2 min as an attempt to draw out any species that had been missed. In muskegs, the design was similar, but instead with 100 × 100 yard (91 × 91 m) plots. Here, the two observers conducted 15‐min point counts. In the meadow habitat, no plots were used, because as Guiguet noted, “… that area being barren apart from the occasional song sparrow, and these frequented the edge for the large part”. Using these point counts, Guiguet concluded that most birds frequented the ocean‐edge of the conifer forest, while the inner edge of the forest, the forest itself, the muskeg, and the open meadows were “practically birdless apart from a few individuals of certain species.” For pelagic species, Guiguet noted that the “total number of trips on pelagic waters was not great” but noted that they recorded every bird seen. He also stated that they would often “chum in” the seabirds by dropping bits of halibut liver, fish, and biscuit crumbs to attract birds to within gunshot range.

For the purposes of this study, we primarily used data from the book published by Guiguet (1953). We derived further context for his work by reading his field notebooks, which were made available to us through the Royal BC Museum in Victoria, BC. We used PlotDigitizer (plotdigitizer.org) to digitize abundance values from figures 18, 19, and 20 (Guiguet 1953). These plots appear to be compiled according to both the data collected on point counts, and all other incidental observations made during the time Guiguet and Martin were on these islands. In cases where a species was not represented in these bar graphs, we drew on data from the written species descriptions and from Guiguet's field notes.

#### 3.2.2 2010 Surveys by CoastWatch Heiltsuk Monitoring and Stewardship

On April 17th, 2010, CoastWatch (led by authors W. Housty and G. Scoville) conducted two systematic surveys: one on the south end of Yáláƛi and the other within the Yáláƛi lagoon. On the south end of Yáláƛi, they established a transect from Yellertlee Bay to the mouth of the lagoon. They counted all birds observed in Yellertlee Bay, along the south shore of Yáláƛi and over to the north edge of Gosling Island, including two open bays. In the lagoon, they observed birds from the mouth of the lagoon to the northern end. At low tide, they surveyed all waterbirds observed on the mudflats, sand, and cobble flats in the lagoon. CoastWatch also opportunistically documented birds while conducting several other wildlife surveys (e.g., for sea mammals, rock cod, deer pellets, amphibians, wolves, beavers, mink, and river otters). In addition, many observations were made during a cultural workshop held on the North Beach in late July 2010, and while transporting personnel and goods to the island. CoastWatch observers were trained and experienced in identifying birds to species level. CoastWatch also conducted mammal surveys, which we use to compare with Guiguet's mammal detections in Table A1.

#### 3.2.3 2011 Surveys by Simon Fraser University Researchers

In 2011, point count stations at 50 locations across the six main islands of Yáláƛi (i.e., Goose, Gosling, Gull, Duck, Snipe, and Swan Island) were selected. These surveys were led by authors R. Field and J. Reynolds. Point count locations were chosen to replicate surveys within the four main habitat types outlined by Guiguet (i.e., climax forest, ecotone, muskeg, meadow, Figure 3a). Points were surveyed three times during the breeding season, between May 12th and June 2nd, 2011. Prior to surveying each point, the date, survey number, start time, point ID, temperature, and weather were recorded. A single observer recorded all birds seen and heard within a 100 m radius of each point for 10 min, separating all observations into minute‐long intervals. The distance between the observer (point) and bird was estimated for each observation. Point counts were conducted within 5 h of sunrise. These protocols were primarily based on Huff et al. (2000) and on our previous detection survey experience on the Central Coast. To obtain an estimate of abundance while avoiding double‐counting individuals, we took the highest count of each species of the three point counts and added that number to the number of individuals detected through casual observations. Casual observations included all bird species (both breeding and migratory) observed outside of the 100 m fixed point count radius and/or allotted 10‐min survey window and assumed not to be duplicate individuals counted during surveys. For the purposes of this study, we used this method to replicate Guiguet's methods as closely as possible, with the assumption that Guiguet also made an effort to avoid double‐counting individuals. However, we recommend that, for future comparisons of birds at Yáláƛi, researchers use the raw point count data (https://doi.org/10.5281/zenodo.11518182). Although, like Guiguet's surveys, these point counting methods targeted forest bird species specifically, anecdotal observations of other bird species, including seabirds, were also recorded. These surveys were conducted with permission granted by the Haílzaqv, and data were shared upon completion of fieldwork and data entry.

#### 3.2.4 2015 Surveys by the Hakai Institute's 100 Islands Project

In 2015, as part of the Hakai Institute's 100 Islands Project, author J. Reynolds and his field crew (led by J. Kennedy) surveyed terrestrial breeding birds on eight islands at Yáláƛi, including Gosling, Duck, Gull, and Snipe Island, as well as four smaller vegetated islands ranging from 0.002 to 0.02 km^2^. Following the 2011 methods described above, 10‐min point count surveys were conducted, where all birds seen and/or heard were recorded. In this case, point counts were spaced at least 250 m apart to maintain independence. Two surveys were conducted at each location: the first in the period between May 7th and 10th, and the second between June 17th and 21st, to account for detection differences in early‐ and late‐season migrant species. Point counts were not conducted during rain or wind speeds above 3 on the Beaufort scale (7–10 km or 12–19 km/h). To minimize interobserver bias, surveyors were selected based on their ability to identify birds of BC, and trial point counts were done to ensure consistency among observers. Casual observations from outside the point counts on this trip were uploaded to eBird. Since we only use presence/absence information about birds from this dataset, we considered all birds observed on point counts, not only those detected within 100 m of the observer. These surveys were conducted with permission granted by the Haílzaqv, and data were shared upon completion of fieldwork and data entry.

#### 3.2.5 eBird

We searched for additional observations of birds at Yáláƛi on eBird, a community science platform. We limited observations to the contemporary period in our study (i.e., 2010–2023). We considered observations from within a 20 km × 20 km grid centered around the Goose Group (eBird 2023). Specifically, we considered birds detected between the latitudes of 52.0114° N and 51.8622° N, and between the longitudes of128.2689° W and 128.6267° W. Birds detected within this 20 km × 20 km square are shaded in red in Table 1. A few species were not present in this square but just outside of it, within 20 km—these are denoted with an asterisk (*) in Table 1. We downloaded the full list of birds in this area from eBird to include species that were missed by Guiguet and our three contemporary surveys. We included detections from the entire year, not just summer months, in Table 1.

**TABLE 1:** Bird species present at Yáláƛi (the Goose Island Archipelago), Heiltsuk (Haíłzaqv) territory, on the Central Coast of British Columbia, Canada, in two time periods.

English name (Haíłzaqvḷa name)	Latin name	1948	2010	2011	2015	eBird
*Ducks, Geese, and Waterfowl: Anatidae*						
Canada Goose (Hṇ́ǧáq)	*Branta canadensis*					
Brant (Haṇx̌‐haṇ́ǧis)	*Branta bernicla*					
Greater White‐fronted Goose	*Anser albifron*					
Mallard (Ns:náq)	*Anas platyrhynchos*					
Northern Pintail	*Anas acuta*	Y				
Northern Shoveler	*Spatula clypeata*					
Green‐winged Teal	*Anas crecca*					
Greater Scaup	*Aythya marila*					
Harlequin Duck	*Histrionicus histrionicus*					
Long‐tailed Duck	*Clangula hyemalis*					
Surf Scoter	*Melanitta perspicillata*					
White‐winged Scoter	*Melanitta deglandi*					
Common Goldeneye (Kvdiṇ)	*Bucephala clangula*					
Bufflehead	*Bucephala albeola*					
Common Merganser (Sx̌ṃ́)	*Mergus merganser*					
Red‐breasted Merganser	*Mergus serrator*					
*Grebes: Podicipedidae*						
Horned Grebe	*Podiceps auritus*					
Red‐necked Grebe	*Podiceps grisegena*					
Western Grebe	*Aechmophorus occidentalis*					
*Pigeons and Doves: Columbidae*						
Eurasian Collared‐Dove (Ha̓ṃ́)	*Streptopelia decaocto*					
*Hummingbirds: Trochilidae*						
Rufous Hummingbird (K̓vák̓vṃt̓a)	*Selasphorus rufus*					
*Cranes: Gruidae*						
Sandhill Crane (H̓dṃ́gvḷí)	*Antigone canadensis*					
*Oystercatchers: Haematopodidae*						
Black Oystercatcher	*Haematopus bachmani*					
*Plovers and Lapwings: Charadriidae*						
Black‐bellied Plover	*Pluvialis squatarola*					
Pacific Golden‐Plover	*Pluvialis fulva*					
Semipalmated Plover	*Charadrius semipalmatus*					
*Sandpipers and Allies: Scolopacidae*						
Greater Yellowlegs (Máx̌vada)	*Tringa melanoleuca*					
Spotted Sandpiper (Máx̌vada)	*Actitis macularius*					
Whimbrel (Máx̌vada)	*Numenius phaeopus*					
Hudsonian Godwit (Máx̌vada)	*Limosa haemastica*					
Marbled Godwit (Máx̌vada)	*Limosa fedoa*					
Ruddy Turnstone (Máx̌vada)	*Arenaria interpres*					
Black Turnstone (Máx̌vada)	*Arenaria melanocephala*					
Wandering Tattler (Máx̌vada)	*Tringa incana*					
Red Knot (Máx̌vada)	*Calidris canutus*					
Surfbird (Máx̌vada)	*Calidris virgata*					
Sanderling (Máx̌vada)	*Calidris alba*					
Dunlin (Máx̌vada)	*Calidris alpina*					
Baird's Sandpiper (Máx̌vada)	*Calidris bairdii*					
Semipalmated Sandpiper (Máx̌vada)	*Calidris pusilla*					
Western Sandpiper (Máx̌vada)	*Calidris mauri*					
Least Sandpiper (Máx̌vada)	*Calidris minutilla*					
Long‐billed Dowitcher (Máx̌vada)	*Limnodromus scolopaceus*					
Short‐billed Dowitcher (Máx̌vada)	*Limnodromus griseus*					
Red Phalarope (Máx̌vada)	*Phalaropus fulicarius*					
Red‐necked Phalarope (Máx̌vada)	*Phalaropus lobatus*					
*Skuas and Jaegers: Stercorariidae*						
Parasitic Jaeger	*Stercorarius parasiticus*					
Pomarine Jaeger	*Stercorarius pomarinus*					
South Polar Skua	*Stercorarius maccormicki*					
*Auks, Murres, and Puffins: Alcidae*						
Common Murre	*Uria aalge*					
Pigeon Guillemot	*Cepphus columba*					
Ancient Murrelet	*Synthliboramphus antiquus*					*
Marbled Murrelet	*Brachyramphus marmoratus*					
Cassin's Auklet	*Ptychoramphus aleuticus*					
Rhinoceros Auklet	*Cerorhinca monocerata*					
Tufted Puffin	*Fratercula cirrhata*					*
*Gulls, Terns, and Skimmers: Laridae*						
Bonaparte's Gull (C̓ígíláǧa)	*Chroicocephalus philadelphia*					
Short‐billed Gull (C̓ígíláǧa)	*Larus brachyrhynchus*					
California Gull (C̓ígíláǧa)	*Larus californicus*					
Herring Gull (C̓ígíláǧa)	*Larus argentatus*					
Glaucous‐winged Gull (C̓ígíláǧa)	*Larus glaucescens*					
Sabine's Gull (C̓ígíláǧa)	*Xema sabini*					
Black‐legged Kittiwake	*Rissa tridactyla*					
*Loons: Gaviidae*						
Red‐throated Loon	*Gavia stellata*					
Pacific Loon	*Gavia pacifica*					
Common Loon (X̌áwí)	*Gavia immer*					
*Albatrosses: Diomedeidae*						
Black‐footed Albatross (Báƛa)	*Phoebastria nigripes*					
*Northern Storm‐Petrels: Hydrobatidae*						
Fork‐tailed Storm‐Petrel	*Hydrobates furcatus*					
Leach's Storm‐Petrel	*Hydrobates leucorhous*					
*Shearwaters and Petrels: Procellariidae*						
Pink‐footed Shearwater	*Ardenna creatopus*					
Flesh‐footed Shearwater	*Ardenna carneipes*					
Short‐tailed Shearwater	*Ardenna tenuirostris*					
Sooty Shearwater	*Ardenna grisea*					
*Cormorants and Shags: Phalacrocoracidae*						
Pelagic Cormorant (C̓ít̓áwí)	*Urile pelagicus*					
Brandt's Cormorant (C̓ít̓áwí)	*Urile penicillatus*					
*Herons, Egrets, and Bitterns: Ardeidae*						
Great Blue Heron (Qáq̓ṇ́c)	*Ardea herodias*					
*Hawks, Eagles, and Kites: Accipitridae*						
Sharp‐shinned Hawk	*Accipiter striatus*					
Northern Goshawk	*Accipiter gentilis*					
Bald Eagle (Wíkv)	*Haliaeetus leucocephalus*					
*Owls: Strigidae*						
Western Screech‐owl	*Megascops kennicottii kennicottii*					
*Kingfishers: Alcedinidae*						
Belted Kingfisher (T̓ást̓am̓ak̓va)	*Megaceryle alcyon*					
*Woodpeckers: Picidae*						
Red‐breasted Sapsucker (λáλap̓ika)	*Sphyrapicus ruber*					
Downy Woodpecker (λáλap̓ika)	*Dryobates pubescens*					
Hairy Woodpecker (λáλap̓ika)	*Dryobates villosus*					
Northern Flicker (λáλap̓ika)	*Colaptes auratus*					
*Falcons and Caracaras: Falconidae*						
Peregrine Falcon	*Falco peregrinus*					
*Tyrant Flycatchers: Tyrannidae*						
Olive‐sided Flycatcher	*Contopus cooperi*					
Western Flycatcher	*Empidonax difficilis*					
*Vireos: Vireonidae*						
Warbling Vireo	*Vireo gilvus*					
*Crows, Jays, and Magpies: Corvidae*						
Steller's Jay (Kváy̓alaqs)	*Cyanocitta stelleri*					
American Crow (K̓áqa)	*Corvus brachyrhynchos*					
Common Raven (Ǧvu̓í)	*Corvus corax*					
*Tits, Chickadees, and Titmice: Paridae*						
Chestnut‐backed Chickadee (Zíziziála)	*Poecile rufescens*					
*Swallows: Hirundinidae*						
Bank Swallow (Mámáɫik̓a)	*Riparia riparia*					
Violet‐green Swallow (Mámáɫik̓a)	*Tachycineta thalassina*					
Barn Swallow (Mámáɫik̓a)	*Hirundo rustica*					
*Kinglets: Regulidae*						
Golden‐crowned Kinglet	*Regulus satrapa*					
Ruby‐crowned Kinglet	*Corthylio calendula*					
*Nuthatches: Sittidae*						
Red‐breasted Nuthatch	*Sitta canadensis*					
*Treecreepers: Certhiidae*						
Brown Creeper	*Certhia americana*					
*Wrens: Troglodytidae*						
Pacific Wren (C̓skṇ́)	*Troglodytes pacificus*					
*Mockingbirds and Thrashers: Mimidae*						
Northern Mockingbird	*Mimus polyglottos*					
*Thrushes and Allies: Turdidae*						
Varied Thrush (Hx̌v:hx̌vṇí)	*Ixoreus naevius*					
American Robin (C̓úp̓álá)	*Turdus migratorius*					
Swainson's Thrush	*Catharus ustulatus*					
Hermit Thrush	*Catharus guttatus*					
*Waxwings: Bombycillidae*						
Cedar Waxwing (Tátix̌as)	*Bombycilla cedrorum*					
*Old World Sparrows: Passeridae*						
House Sparrow	*Passer domesticus*					
*Wagtails and Pipits: Motacillidae*						
American Pipit	*Anthus rubescens*					
*Finches, Euphonias, and Allies: Fringillidae*						
Red Crossbill	*Loxia curvirostra*					
Pine Siskin	*Spinus pinus*					
*New World Sparrows: Passerellidae*						
Savannah Sparrow	*Passerculus sandwichensis*					
White‐crowned Sparrow	*Zonotrichia leucophrys*					
Golden‐crowned Sparrow	*Zonotrichia atricapilla*					
Fox Sparrow	*Passerella iliaca*					
Song Sparrow	*Melospiza melodia*					
Lincoln's Sparrow	*Melospiza lincolnii*					
Dark‐eyed Junco	*Junco hyemalis*					
*Troupials and Allies: Icteridae*						
Brown headed Cowbird	*Molothrus ater*					
*New World Warblers: Parulidae*						
Orange‐crowned Warbler	*Leiothlypis celata*					
Common Yellowthroat	*Geothlypis trichas*					
Yellow Warbler	*Setophaga petechia*					
Yellow‐rumped Warbler	*Setophaga coronata*					
Townsend's Warbler	*Setophaga townsendi*					
Wilson's Warbler	*Cardellina pusilla*					
*Cardinals and Allies: Cardinalidae*						
Western Tanager	*Piranga ludoviciana*					

*Note:* Detections made by Charles J. Guiguet in the earlier time period (1948) are shaded dark red. Contemporary detections are in light red, and come from CoastWatch surveys (2010), a follow‐up study led by Simon Fraser University researchers (2011), the Hakai Institute's 100 Islands Project (2015), and detections uploaded to the community science platform, eBird (2010–2023). An asterisk (*) denotes that the observation is from further than 20 km from Goose Island but within 60 km. Birds are listed in taxonomic order as presented by the Cornell Lab of Ornithology. Some Haíłzaqvḷa names refer to larger taxonomic groups of birds (e.g., Máx̌vada refers to birds in family Scolopacidae).

### 3.3 A Note on Taxonomy

Several name changes and taxonomic revisions have occurred since Guiguet's time at Yáláƛi. As a result, we have converted several historical names to their current equivalents. Arctic Loon (*Gavia arctica*) and Pacific Loon (*G. pacifica*) were split in 1985—thus, we include Guiguet's reported Arctic Loons as Pacific Loons. Western Flycatcher was split into Cordilleran Flycatcher (*Empidonax occidentalis*) and Pacific‐slope Flycatcher (*E. difficilis*) in 1989, which were recombined in 2023. Thus, we refer to this species as Western Flycatcher. Pacific Wren (*Troglodytes pacificus*) split from Winter Wren (*T. hiemalis*) in 2010. Northwestern Crow (*Corvus caurinus*) and American Crow (*C. brachyrhynchos*) were recombined to American Crow in 2020. Short‐billed Gull (*Larus canus*) was commonly known as “Mew Gull” until 2021. What are now known as Short‐tailed Shearwater (*Ardenna tenuirostris*) were termed “Slender‐billed Shearwater” in the past, modern Flesh‐footed Shearwater (*Puffinus carneipes*) were known as “Pale‐footed Shearwater,” Leach's Storm‐petrels (*Hydrobates leucorhous*) were known as “Boreal Petrels,” and Long‐tailed Ducks (*Clangula hyemalis*) were historically called “Old Sq**w,” a name which is particularly outdated and inappropriate due to its racist connotations. Peregrine Falcon (*Falco peregrinus*) was called “Duck Hawk,” and Sooty Grouse (*Dendragapus fuliginosus*) was known as “Blue Grouse.” The Oregon subspecies of Dark‐eyed Junco (*Junco hyemalis oreganus*) were known as Oregon Junco (*Junco oregonus*).

### 3.4 Historical Versus Contemporary Comparisons

**FIGURE A3:**
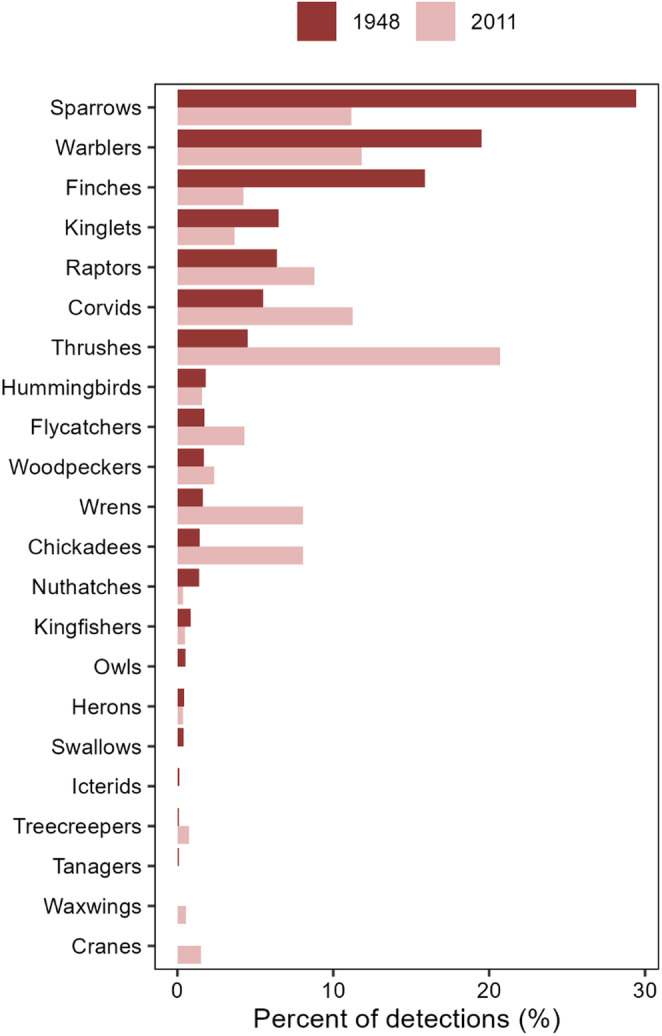
Forest bird species relative abundances (percent of detections) observed by C.J. Guiguet in 1948 and through systematic surveys conducted through Simon Fraser University in 2011, binned into guilds.

Given that we have no quantification of search effort for surveys conducted in the historical period, we have only made comparisons of species relative abundances between the historical and contemporary periods. First, because our 2011 SFU surveys were designed to support a historical and contemporary comparison, we targeted the same habitat types (i.e., forest, muskeg, transitional habitats, meadows) as Guiguet targeted in 1948. As such, we were able to calculate proportional relative abundances of all birds detected on the semi‐systematic surveys in each of these two studies. We have also calculated the relative proportions of different guilds of these birds (Figure A3).

For all other species, given that we have no quantification of effort and sometimes also no quantification of observed abundance in Guiguet's surveys, we combined the species detected in any of the five data sources into a table and noted whether each species was observed or not by each source (Table 1).

### 3.5 Our Approach

Overall, our approach is rooted in Heiltsuk knowledge, which, like other Indigenous knowledges, cannot be fully captured or evaluated through a solely Western scientific framework. We acknowledge that ambiguity may result from a multidimensional perspective that contrasts with the detailed focus of conventional ecological measurements. Some aspects of Heiltsuk knowledge, particularly regarding Yáláƛi, are sensitive and are only shared in general terms. Incorporating this knowledge into research supports broader goals, including the recognition and respect of Indigenous ways of knowing both traditional and contemporary, aligning with reconciliation efforts.

## 4 Results

**FIGURE 4:**
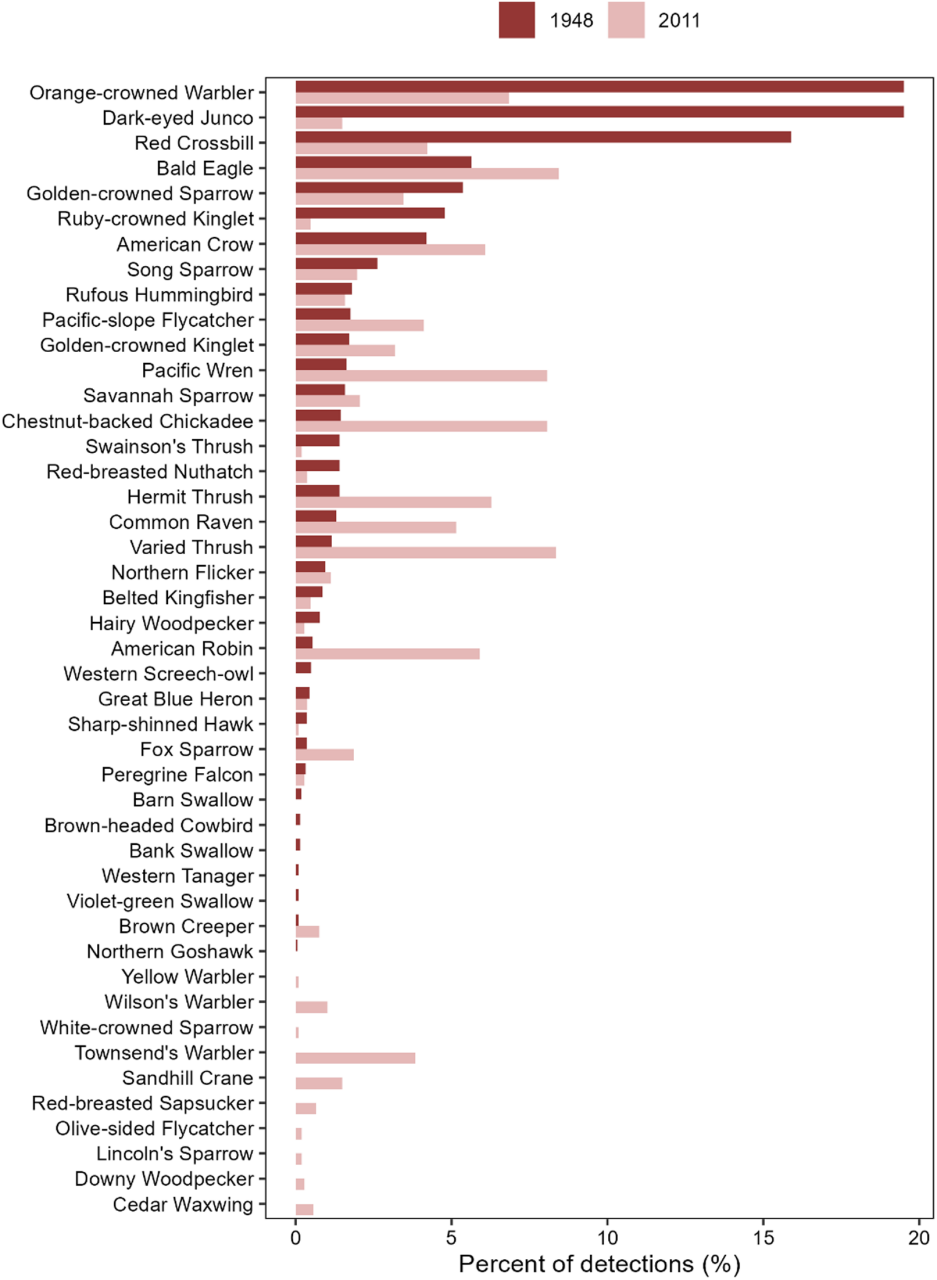
Forest bird species relative abundances (percent of detections) observed by C.J. Guiguet in 1948 and through systematic surveys conducted through Simon Fraser University in 2011.

There was pronounced variation between bird communities present at Yáláƛi from 1948 compared with the present day (Table 1). Observations of the most noteworthy species are described in the appendix. We also found several differences in forest bird communities between Guiguet's observations in 1948 and ours in 2011 (Figure 4). In 1948, three species dominated in terms of relative abundance: Orange‐crowned Warbler, Dark‐eyed Junco, and Red Crossbill. These three species comprised 55% of all detections. In 2011, these three species made up only 13% of all detections. Additionally, in 2011, the top three most‐recorded species were Bald Eagle, Varied Thrush, and Pacific Wren. In total, these three species made up only 25% of all detections, suggesting a much more even distribution in the relative abundance of species in the latter time period. Additionally, in 1948, the only species of warbler detected was Orange‐crowned Warbler, while the 2011 survey additionally found comparatively high relative abundances of Townsend's Warbler and Wilson's Warbler. Furthermore, despite being tied for highest relative abundance in 1949, where it made up roughly 20% of all detections, the Dark‐eyed Junco was only 20th most abundant in 2011, where it made up less than 2% of all detections. The reverse is true for Varied Thrush. In 2011, this species was the second most abundant, comprising 8% of all detections. However, in 1948, this species was 19th most abundant, and accounted for only 1% of all detections. Pacific Wren and Chestnut‐backed Chickadee were also very abundant in 2011 (third and fourth most abundant), totaling over 16% of detections in 2011. Together, these species only accounted for 3% of detections in 1948.

## 5 Discussion

In our comparison of the birds present at Yáláƛi (Goose Island) in 1948 with those detected more recently, we observed some notable differences in bird communities between time periods. Here, we discuss possible reasons for these changes, drawing from a diversity of sources including insight from local and Indigenous knowledge holders, local and regional experts on both terrestrial and marine birds, and fellow bird enthusiasts. Our intent is to contextualize and deepen our understanding of the natural variation in species communities at Yáláƛi, and to predict how these communities may respond to global change. While there are likely Yáláƛi‐specific drivers, we speculate that changes in species communities here may be representative of what might also be occurring elsewhere in the region. We hope that this paper will be useful for future comparisons of bird communities. We also discuss limitations of the data collected and compare our findings with what is known to be true by members of the community who have extensive personal experiences with this landscape. We can think of three potential reasons for changes in bird communities, including changes in the social–ecological landscape, changes in weather and/or climate, and interannual variability.

### 5.1 Changes in the Social–Ecological Landscape

Over the 75‐year period covered by this study, the landscape of Yáláƛi has not been affected by logging, unlike most of the rest of British Columbia. Instead, it has been shaped by other changes in human use of the islands, changes in deer and wolf communities, and large‐scale natural phenomena, including tsunamis and marine heat waves. At first glance, when looking at aerial imagery, it appears that both the extensive bog area and the meadow habitat may be reduced in 2023 (Figure 3d), in comparison with Guiguet's hand‐drawn depiction of the vegetation in 1948 (Figure 3a). In Guiguet's map, the bog habitat seems to have extended down toward the ocean at two distinct points on the eastern side of the island. However, when compared to an aerial photo taken in 1955 (Figure 3b), we can observe a similarly forested area mid‐island. Large‐scale changes in forest structure are unlikely to occur on the timescale of 75 years (Brown and Hebda 2002; Walsh et al. 2015). Accordingly, we discount the possibility of a distinctly different bog versus forest composition.

Drastic changes in the mammal community on Yáláƛi over the last 75 years, however, have had profound effects on understory vegetation, with large potential impacts to forest bird communities. Despite the attention Guiguet paid to mammals (see his species list in Table A1), he makes no mention of the two mammal species Yáláƛi is locally known for—Black‐tailed Deer (*Odocoileus hemionus columbianus*) and Gray Wolves (*Canis lupus*). It seems highly likely, therefore, that there were no deer present on the Goose Islands during Guiguet's time there. Indeed, early local stories (1930s, 1940s) mention dogs left on the island seen digging clams to survive, supporting the idea that there were no deer present on Yáláƛi prior to mid‐century (L. Jorgenson, personal communication). Similarly, Guiguet mentioned that abandoned dogs were observed on the island in 1904, citing a personal communication with sealer and fisherman M. Lohbrunner from 1948 (Guiguet 1953). Just a few decades later, Yáláƛi became the “go to” hunting spot for deer, with hunters harvesting up to 12 deer in the lagoon alone at low tide in a couple of hours. However, by 2000, and in the absence of wolves, there were so many deer that hunting activities ceased due to poor body condition and high tick infestations (L. Jorgenson, personal communication). As noted by CoastWatch staff, the remaining vegetation post‐deer consisted predominantly of large, old cedar trees, with a notable lack of cedar saplings and other understory shrubs and herbs—a common ecological state when deer are free from predation in temperate rainforests (Chollet et al. 2021).

The hyper‐abundance of deer can have long‐lasting, severe impacts on songbird communities by decreasing the amount of suitable habitat (Pojar 2008; Martin, Arcese, and Scheerder 2011), increasing their predation risks, and altering the availability of invertebrates prey items (Martin, Allombert, and Gaston 2008). Deer browsing reduces the availability of essential nesting and feeding habitats through cover reduction and simplification of shrub architecture, which also results in increased nest exposure (Martin, Allombert, and Gaston 2008). As a result, deer‐free islands host far more diverse and abundant bird communities than those with high deer density (Martin, Arcese, and Scheerder 2011). The life histories of different songbird species make them variable in their sensitivities to these pressures. For instance, Fox Sparrows, Song Sparrows, Wilson's Warblers, Orange‐crowned Warblers, Rufous Hummingbirds, Swainson's Thrushes, Hermit Thrushes, Varied Thrushes, and Pacific Wrens are highly dependent on understory structures for nesting and foraging, while Pacific‐slope Flycatchers, Dark‐eyed Juncos, Common Ravens, Brown Creepers, and Chestnut‐backed Chickadees are more reliant on other forest habitats, including the forest canopy, for feeding and nesting and therefore less likely to experience adverse deer impacts (Martin, Allombert, and Gaston 2008; Martin, Arcese, and Scheerder 2011; Chollet et al. 2015). We found that these predictions hold true for some species at Yáláƛi; prior to deer appearing on the islands, Guiguet detected higher relative abundances of Orange‐crowned Warblers, Song Sparrows, Rufous Hummingbirds, and Swainson's Thrushes than in our 2011 surveys (Figure 4). Likewise, Pacific‐slope Flycatchers, Common Ravens, Brown Creepers, and Chestnut‐backed Chickadees had higher relative abundances in 2011, suggesting that these species may be less sensitive to deer impacts. However, despite deer presence, we found higher relative abundances of certain understory species, including Pacific Wrens, Hermit Thrushes, Varied Thrushes, and Fox Sparrows in 2011 than Guiguet found during the deer‐free period. Some of these species, particularly Pacific Wrens and Varied Thrushes, are known to prefer older forests, and thus, their increased relative abundance over time may be due in part to increasing forest age (George 2020; Toews and Irwin 2020). Interestingly, Dark‐eyed Juncos are one of the few species known to thrive on islands with high deer density (Martin, Arcese, and Scheerder 2011) but had far higher relative abundance during Guiguet's deer‐free surveys in 1948 than in 2011 (20% of detections vs. 2%), suggesting that there must have been a different driver for their high abundances in 1948.

Sometime after 2004, when the island was surveyed for wolves (Darimont et al. 2004) and prior to CoastWatch surveys in 2010, there was yet another dramatic shift in the mammal community. Local community members spotted wolves entering Golby Pass, in the McMullin Group archipelago, which is the shortest route to Yáláƛi from the east. Wolf signs and sightings appeared in 2007, and they evidently became abundant on the islands and reproduced; author J. Reynolds saw footprints on the large lagoon in 2011 that included those from pups, and author R. Field's field crew observed wolves on Goose, Gosling, and Duck Islands in that year. Likely subsidized by extensive marine resources (Roffler et al. 2023), the wolves imposed a pronounced reduction in deer populations. In more recent years, deer appear to have completely disappeared, or at least, have been reduced back to very low abundances at Yáláƛi (L. Jorgenson, personal communication). The understory has begun to make a resurgence, with the potential to influence bird communities again in coming years.

Changes in the occurrences of other mammals may also have influenced Yáláƛi vegetation structure and thus bird communities. Some mammals known to be ubiquitous in the region were evidently missing from Yáláƛi in the past. C.J. Guiguet mentioned: “For some inexplicable reason, mink and otter apparently do not occur upon the Goose Islands.” In this statement, he refers to the American mink (*Neogale vison*), and the North American river otter (*Lontra canadensis*). These two species, especially the river otter, can have large impacts on terrestrial vegetation, both through a combination of nutrient deposition and disturbance (Roe et al. 2010; Obrist et al. 2022). This compound effect has been shown to influence terrestrial breeding bird communities, including on islands near the Goose Group Islands on the Central Coast of BC, where Obrist et al. (2020) found fewer bird species on islands with higher levels of δ^15^N, a metric that roughly indicates river otter activity (Ben‐David et al. 1998). The reason for this decrease in species richness did not appear to be from competition for nutrients, but may indicate the lack of tolerance by certain species to conditions created by river otter activity (Obrist et al. 2023). Although Guiguet did not observe them during his stay in the area, all three contemporary surveys observed evidence of both river otter and mink activity at Yáláƛi. Both of these mammals are opportunistic predators known to prey on birds. The surveys in 1948 were characterized by a large representation Dark‐eyed Juncos, which nest on the ground (Nolan Jr. et al. 2020), and Orange‐crowned Warblers, which nest on or near the ground (Gilbert, Sogge, and van Riper 2020). Although we have not tested this experimentally, it appears that in the presence of these mammals in the contemporary period, there are relatively fewer ground nesting species present than were historically present.

Changes in human use of the islands is another plausible reason for changes in bird communities at Yáláƛi. Human traffic to Yáláƛi has decreased over the last two decades since the deer populations declined (W. Housty, personal observation). Previously, the island and the surrounding waters served as a multifaceted harvesting ground for many culturally important species: seaweed, geese, ducks, deer, halibut, and cod (Heiltsuk Traditional Use Study, Heiltsuk Nation 1998‐present). Although Heiltsuk community members consistently identify Yáláƛi as one of the most important remaining halibut fishing areas in the territory (W. Campbell Jr., personal communication), recent declines in halibut and cod (in concert with expensive fuel prices) have further contributed to reduced human activity on and around the islands. Before the 1920s1940s, the entire village of Wágļísļa (Bella Bella) would travel to Yáláƛi for the summer (Heiltsuk Traditional Use Study, Heiltsuk Nation 1998‐present). Furthermore, prior to European colonization and associated disease epidemics, Haíłzaqv populations were much larger. There would have been more impact on certain bird populations then, when there were large villages located at Yáláƛi (L. Jorgenson, personal communication). Traditional Use Studies conducted by Haíłzaqv Tribal Council with community elders in the 1990s indicated that historically, ducks and geese were commonly harvested in many places around the islands, although most efforts focused on the lagoon (Heiltsuk Traditional Use Study, Heiltsuk Nation 1998‐present). Although difficult to discern with the data available, L. Jorgensen suggested that duck and goose populations have experienced localized population decreases in recent years, most significantly in the lagoon area, where there was once a hunting cabin. L. Jorgensen also speculated that there may have been changes to food sources in the lagoon, stating that the decline of ducks and geese was noted in the community prior to the introduction of wolves, suggesting depletion by or avoidance of predators do not explain the apparent change. Neither Guiguet's surveys nor our modern ones detected changes in goose and duck abundances at Yáláƛi overall, but this may the artifact of a mismatch in both the location and timing between surveys and traditional harvest practices. In addition, the modern surveys (aside from CoastWatch) focused on terrestrial species, without intentional overlap with culturally important areas. Thus, local knowledge provides a finer spatial resolution of goose and duck occurrences than our surveys, suggesting that these species have shifted from away from using the lagoon (W. Housty and L. Jorgensen, personal communication).

Human‐related fire activity has likely contributed to changes in habitat structure and vegetation composition of Yáláƛi over the past century. This is evidenced by the presence of more fire‐scarred trees and distinct vegetation communities at past‐habitation sites, including those on Gosling Island, compared to control sites (Hoffman, Lertzman, and Starzomski 2017). From 1376 to 1893, regular low‐ to moderate‐severity fires coincided with Indigenous habitation, with no fire activity recorded after this period, likely due to forced relocation to settlements (Hoffman, Lertzman, and Starzomski 2017). The absence of Indigenous fire stewardship since then has contributed to changes in vegetation structure and local biodiversity (Hoffman et al. 2021). While large stand‐clearing fires are unlikely at Yáláƛi, post‐fire vegetation responses can be slow, even following low to moderate severity fires. Many plant species in these perhumid temperate rain forests are poorly adapted to fire disturbance and may not survive or recolonize for a long time post‐fire (Banner et al. 2005), leading to persistent changes in understory plant communities. Regular burning historically promoted the regrowth of Western Redcedar (*Thuja plicata*) near habitation sites (Turner 1999), a tree species negatively associated with some bird species like the Pacific Wren (Holmes and Robinsoni 1988; Wilcox, Wagner, and Reynolds 2021), a species which we found to be relatively more common in the contemporary period of this study. The gradual shift in vegetation composition and structure due to the absence of regular burning has likely influenced habitat availability and suitability for various bird species over the past 130 fire‐free years.

Finally, physical changes in landscape from large‐scale natural phenomena likely had an impact on the bird communities of Yáláƛi. Prior to 1964, author W. Housty's late grandfather recalled a fine, white sand in the Goose Island lagoon, which packed down hard during low tide. This offered the ideal foundation for big soccer tournaments. Although often associated with shell middens, the white sand in the lagoon is likely the result of weathering bedrock (B. Menounos, personal communication). In 1964, a 9.2 magnitude megathrust earthquake in Alaska caused a tsunami that changed the structure of the lagoon (United States Geological Survey n.d.). The tide was high during the earthquake, and consequently when the water was drawn out of the lagoon the sand left with it, leaving behind a rockier, muddier habitat. Some remnants of white sand remain at the mouth of the lagoon. Several local accounts corroborate the occurrence of a rapid fall and subsequent rise of sea levels, with many boats getting momentarily stranded, high and dry (Heiltsuk Traditional Use Study, Heiltsuk Nation 1998‐present). The resulting transition from sandy to rocky and muddy shores may have impacted which bird species and/or prey items were able to use resources in the lagoon. It is thought that this reshaping of the lagoon may have impacted the availability of harvest species in the area (i.e., geese and ducks, Heiltsuk Traditional Use Study, Heiltsuk Nation 1998‐present).

### 5.2 Changes in Weather, Climate, and Frequency of Extreme Events

**FIGURE A4:**
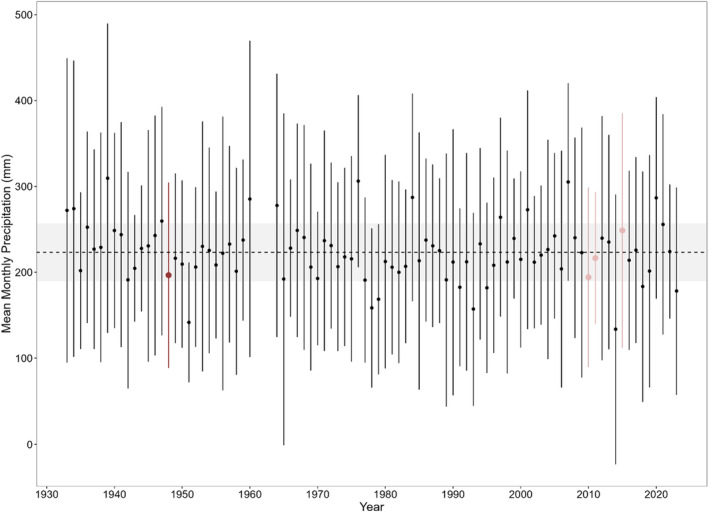
Mean monthly precipitation (mm) from 1933 to 2023, taken from weather stations in Bella Bella, BC. Data were summed across months and averaged across years. Dots represent annual means, and bars represent one standard deviation. The black dashed line and shaded area represent the mean and one standard deviation of the entire dataset. The dark red dot and lines represent 1948, the year of Guiguet's surveys. The data points highlighted in lighter red represent 2010, 2011, and 2015—the years for which we present contemporary bird survey data in this study. Data for 1933–1977 come from station ID 378 (climate ID 1060810), for 2006–2011, the data come from station ID 44766 (climate ID 1060811), and for 2012–2023, the data are from station ID 49388 (1060815), all in Bella Bella, BC. The data for 1978–2005 come from the lighthouse at Dryad Point, BC, 3.4 km east of Bella Bella (Climate ID 1062544).

**FIGURE A5:**
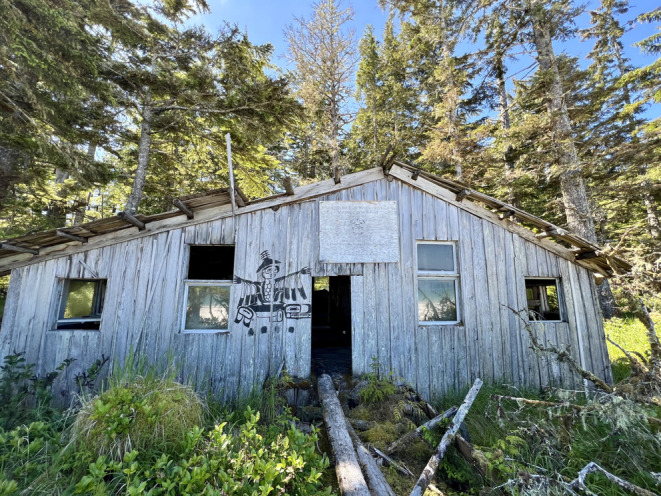
Old hunting cabin near the lagoon on Goose Island (Yáláƛi), British Columbia, Photo by Chris Darimont.

Climatic changes also provide a likely reason for variability in the bird communities detected at Yáláƛi in 1948 and in more recent years. Changes in weather, climate, and the intensity and frequency of extreme weather events are occurring globally (National Academies of Sciences, Engineering, and Medicine 2016), impacting migratory birds on both their breeding and wintering grounds (Robinson et al. 2009). Most recently, 2023 was the hottest year on record globally (World Meteorological Organization 2023). Guiguet noted that 1948 was also a particularly warm and dry year (Guiguet 1953). As such, it is somewhat unclear whether the account of bird communities we have from him provides an accurate representation of bird species compositions in the area in that time period. In his 1953 work, he stated, “The climate was indeed excellent” and, “Rain approaching across Queen's Sound was an unusual sight.” Finally, he noted that “Whether these conditions were atypical is difficult to say in the light of inadequate knowledge.” According to data from weather stations in Wágļísļa (Bella Bella), it was atypical for rain to be “unusual” on this part of the coast (Figure A4). The early summer of 1948 was particularly dry: the April, May, and June rainfalls (48 mm, 93 mm, and 49 mm) were far below monthly averages for the area (198 mm ± 82 mm, 158 mm ± 79 mm, and 145 mm ± 72 mm). This may have influenced which species occurred at Yáláƛi during those summers. For example, there is evidence (e.g., Kirk et al. 1996) that Dark‐eyed Juncos tend to prefer drier habitats. This could explain why Guiguet observed such a high relative abundance of this species in a drier than usual year, as compared to our 2011 surveys, which represented a more average year in terms of precipitation on the Central Coast (Figure A5).

### 5.3 Interannual Variability

For many bird species, there can be a lot of variability in abundance from year to year, so it is difficult to make conclusions based on any one particular year. This highlights the importance of long‐term monitoring of an area. For instance, irruptive species, such as Red Crossbills, closely follow the distribution patterns of annually varying conifer seed crops (Koenig and Knops 2001). Guiguet observed just a single warbler species in 1948—Orange‐crowned Warbler. Meanwhile, contemporary surveys show that at least two additional species, Townsend's Warbler and Wilson's Warbler, are relatively common. Without more data, we are unable to speculate as to whether these patterns in species presence were simply a result of stochasticity or a mechanistic cause.

#### 5.3.1 Challenges With This Dataset and Future Recommendations

Using historical ecological data to answer pertinent ecological questions can be difficult due to differences in methodology, reporting, and changing objectives and perspectives throughout time. For example, sampling effort (Reznick, Baxter, and Endler 1994), weather, sampling methods, and surveyor experience can all greatly influence estimates of populations, and must be carefully documented if the intention is to make meaningful comparisons with future data (Shaffer, Fisher, and Davidson 1998).

One of the issues we encountered when planning this study was that Guiguet made no consistent record of sampling and search effort, and often did not record accurate locations of observations. We were able to glean some of this information from his field notebooks. However, this lack of data made it unfeasible to implement any direct comparisons in the abundances of species observed. As such, we have made several recommendations in service of future interest in continuing to monitor bird populations at Yáláƛi.

In the future, we recommend surveyors use a standardized field protocol, such as the point counts used in our 2011 and 2015 field studies, with predetermined survey locations representative of the various habitat types on the islands. For terrestrial species, this will likely involve repeating fixed‐time point count surveys in each of the habitat types described by Guiguet: muskeg, meadow, ecotone‐type forest, and climax forest (Figure 3a,c). For a more accurate account of marine bird species, fixed‐length transect surveys should be conducted in areas of interest. Given that there are differences between early‐ and late‐season migrants in this area, and even into the fall, we recommend surveys be done once in the early spring, once in late spring/early summer, and—if possible—again in the fall. If the goal is to track changes in species abundance, keeping track of survey effort is critical. As such, we recommend conducting fixed time point count surveys, documenting numbers of observers present, and noting weather and noise conditions. If the goal is to track changes in presence over time, then enlisting citizen science initiatives through platforms like eBird and iNaturalist may be an affordable complement that minimizes required resources. We recommend repeating surveys on a 20‐year rotation at minimum. This would make surveys comparable to those of the BC Breeding Bird Atlas, which occurred from 2008 until 2012. If this Atlas is repeated on a 20‐year cycle, it could provide a powerful comparison of changes in bird abundance over time. The same approach would be beneficial if implemented at Yáláƛi.

While we do not claim to present a clear narrative for the precise drivers of change in bird communities at Yáláƛi over the last 75 years, we hope that it is apparent that despite lack of pressure from logging, urban development, and other industrial activities, bird communities here are changing. Thus, we strongly recommend continued efforts to monitor the bird communities at Yáláƛi to understand what birds are present in the area, whether and why their communities are shifting over time, and to continue building a biodiversity baseline to evaluate the impacts of future threats. We hope that continued Haíłzaqv use of the area and its resources will yield further insights that can be combined with historical and future surveys.

## Author Contributions

**Debora S. Obrist:** conceptualization (supporting), data curation (supporting), formal analysis (lead), writing – original draft (lead). **Elizabeth Jane Pendray:** data curation (equal), formal analysis (equal), methodology (supporting), writing – review and editing (equal). **William Housty:** conceptualization (equal), data curation (equal), writing – review and editing (equal). **Rachel D. Field:** conceptualization (equal), data curation (equal), methodology (equal), writing – review and editing (equal). **Gerald W. Scoville:** conceptualization (equal), data curation (equal), writing – review and editing (equal). **Allison M. Dennert:** writing – review and editing (equal). **Chris T. Darimont:** writing – review and editing (equal). **John D. Reynolds:** conceptualization (equal), funding acquisition (lead), methodology (equal), writing – review and editing (equal).

## Conflicts of Interest

The authors declare no conflicts of interest.

## Data Availability

Charles C. Guiguet's data are available in his 1953 publication. The 2010 CoastWatch survey data are represented in Table 1 of this manuscript, but further details about specific taxa can be requested from author J. Scoville directly (jerry.scoville@gmail.com). Data for the 2011 Simon Fraser University surveys are available at https://doi.org/10.5281/zenodo.11518182, and 2015 100 Islands surveys are available at https://doi.org/10.21966/10tk‐4956.

## Appendix A

*Noteworthy observations*

The following observations are those which we thought to be worthy of special mention.

### A.1. Ducks, Geese, and Waterfowl: Anatidae

The most noteworthy waterfowl species are those that are important for traditional harvest practices in Haíłzaqv territory. These include Common Goldeneye, Canada Goose, Surf Scoter, White‐winged Scoter, Common Merganser, and Mallard (H. Humchitt, personal communication). Based on the Traditional Use Studies conducted in the 1990s, ducks and geese were once very abundant on Yáláƛi. Although they were harvested throughout the archipelago, efforts were once concentrated in the lagoon area of Goose Island.

Canada Geese appeared to be abundant in the area in 1948, and Guiguet mentioned many fly‐overs. In 2010, CoastWatch reported several flocks on the island, with the largest flock consisting of 77 birds. SFU researchers also detected Canada Geese during their stay on the islands in both 2011 and 2015. Many records exist on eBird.

Guiguet reported a small number of Mallards on muskeg ponds and stated that a Mr. T. Balmer, from the nearby township of Ocean Falls, had reported that Mallards were fairly numerous in the fall. CoastWatch reported Mallards as the most abundant duck on Goose Island, reporting flocks throughout spring migration, including a flock of 274 birds on April 17th, 2010, in the lagoon. Mallards were recorded by both groups of SFU researchers. Eight individuals were recorded by J. Reynolds on eBird.

Guiguet reported several hundred Surf Scoters passing by the islands, with approximately 30 resting at the south end of the main island. Our research team recorded 29 Surf Scoters in 2011, and many Surf Scoters have been recorded in the area on eBird.

As for mergansers, Guiguet reported rare sightings of both American (now Common) Merganser, and Red‐breasted Merganser. CoastWatch reported some sightings of both species; 11 Common Mergansers and eight Red‐breasted Mergansers on April 17, 2010. In 2011, SFU researchers reported 29 sightings of Common Merganser. On eBird, J. Reynolds recorded four Common Merganser individuals twice on Swan Island, on May 24 and May 27, 2011, as well as one individual in the water off Snipe Island on May 21, 2011. B. Judson recorded three individuals at the south end of Goose Island on June 19, 2022. B. Whittington has the only eBird record of Red‐breasted Mergansers; two individuals were recorded on May 17, 2010.

Guiguet recorded 10 Common Goldeneye ducks on May 11th, and another individual on May 20, 1948.

### A.2. Grebes: Podicipedidae

Guiguet commented in the publication of his 1948 observations that it was noteworthy that no grebes were recorded during his stay on the islands. This absence contrasts with abundant sightings in the contemporary period. Red‐necked Grebes were detected most commonly, with three individuals observed by CoastWatch in 2010, and multiples by eBird observers: 10 records by J. Reynolds in 2010, 2011, and 2015, totaling 10 individuals sighted from the northern point of Yáláƛi, five individuals from Duck Island, 10 from Snipe Island, and three from Gosling Island, two records of 10 individuals by B. Starzomski in 2015—one record north of Gosling Island and one from Snipe Island, and two records of three individuals by K. Clauston in 2015. Two Western Grebes were also observed by the SFU research team in 2011. On eBird, 2 Horned Grebes were detected by B. Starzomski in 2015.

### A.3. Sandhill Crane—*Antigone canadensis*

Guiguet recorded hearing “a sizable flock” of cranes on August 15, 1948, but he neither saw them nor did he estimate the size of the flock for this lone record. No individuals were detected in a 2006 helicopter survey specifically searching for nests and nesting individuals of this species from the air (Roessingh and Penn 2010). However, in May 2010, CoastWatch observed a pair and a trio of cranes flying into the interior of Goose Island, and in July 2010, observed a pair and a trio feeding in the lagoon. The SFU research team recorded a total of 16 Sandhill Cranes between their systematic surveys and casual observations in May and June 2011. The 100 Islands Project team in 2015 also recorded observations of this species. Additionally, this species has been recorded many times on eBird. This species has been consistently present seasonally on the islands, though breeding has not been confirmed. Local opinion is that Sandhill Cranes may be becoming more common in Haíłzaqv territory generally, or at least, that people are becoming more aware of them (L. Jorgenson, personal communication).

### A.4. Godwits

#### A.4.1. Hudsonian Godwit—*Limosa haemastica*

A single individual of this species was observed on the north beach of Gosling Island, by R. Field, as part of the SFU research team in 2011.

#### A.4.2. Marbled Godwit—*Limosa fedoa*

The only records for this species come from eBird, with three observations of a single individual detected on the south end of Goose Island by both B. Whittington in July 2010 and K. Clouston in July 2015, and one detected by B. Starzomski on Snipe Island in July 2015.

### A.5. Seabirds

#### A.5.1. Parasitic Jaeger—*Stercorarius parasiticus*

Guiguet observed two individuals 15 “miles” (unclear if referring to statute or nautical miles, approximately 24 km) west of the island on June 10, and collected another individual 19 km north of Yáláƛi on August 14, 1948, which was found to have been eating sand lance. No other surveys have observed this species near the Yáláƛi, though pelagic surveys recorded in eBird have detected this species ~30 km west of the islands (S. Landes and J. Hammond in 2019).

#### A.5.2. Pomarine Jaeger—*Stercorarius pomarinus*

Guiguet recorded one individual 29 km west of Yáláƛi on June 13, and three birds on July 13, 1948, two of which were collected and “proved to be females, both showing signs of brood patches recently ‘downed over’” (Guiguet 1953).

#### A.5.3. South Polar Skua—*Stercorarius maccormicki*

This species was observed three times by Guiguet, on June 13 and June 27, 1948, 29 km west of Yáláƛi, and on August 17, 1948, 19 km to the south. Collected specimens were found to have been feeding on entrails of trolled coho salmon (*Oncorhynchus kisutch*), and on sand lance (*Ammodytes* sp.). The nearest modern observations of this species on eBird are located ~40 km west of Yáláƛi.

#### A.5.4. Black‐Footed Albatross—*Phoebastria nigripes*

Guiguet observed this species regularly in small numbers on the Goose Island Banks, including feeding on offal discarded by halibut fishermen in May 1948. He noted that the species “usually foraged beyond a point 10–18 miles (16–29 km) off the coast but on foggy mornings and in periods of very rough weather individuals were sometimes sighted from the shore” (Guiguet 1953). The nearest modern observations of this species on eBird are located ~20 km southwest of Yáláƛi.

#### A.5.5. Fork‐Tailed Storm‐Petrel—*Hydrobates furcatus*

This was the second most common pelagic species observed by Guiguet in 1948, after Sooty Shearwaters. From May through August, this species was numerous on the Banks, though sometimes “appearing about the islands in numbers during the night and on foggy days”. As Guiguet regularly observed individuals flying seaward in the mornings, he attempted to locate the breeding location; there was no evidence of breeding at Yáláƛi, and though he did find a small colony on Fingal Island (~8 km north of Goose Island), “this could hardly account for the large numbers observed at sea, samples of which invariably carried brood patches” (Guiguet 1953). In late July and August, Guiguet observed a decline in the number of this species on the banks, but they began to be observed east and north of the islands near Hunter Island (~13 km away), and further inland and north in Mathieson Channel, the mouth of which lies approximately 35 km to the north, and Kynoch Inlet, which branches out from Mathieson Channel at roughly 85 km north of Yáláƛi. These inland observations occurred around salmon fishing seine boats, and Guiguet commented, “whether this is due to an abundance of food made available by commercial fishing operations, or whether it is intrinsic behaviour, is unknown at present.” eBird observations of this species from the area include a single individual detected by R. Cannings in 2010 and another by M. Jackson in 2020. H. Carolsfeld observed approximately two to five individuals of this species in 2016 (H. Carolsfeld, personal communication).

#### A.5.6. Leach's Storm‐Petrel—*Hydrobates leucorhous*

Guiguet observed this species only twice and noted that it is generally found west of the Goose Group. One individual was observed 19 km west of Gosling Rocks on June 26, 1948, and the second was collected at night between Goose Island and Gosling Rocks on July 25, 1948. The collected specimen was a male with a bare brood patch, and Guiguet inferred that it was nesting near the capture location. Most eBird observations are also from west of the islands, but there was a single individual detected by R. Cannings in 2010, near the eastern shore of Goose Island.

#### A.5.7. Black‐Vented Shearwater—*Puffinus opisthomelas*

Guiguet reported a single individual 29 km southwest of the Goose Islands on August 14, 1948. His field notes indicate some degree of uncertainty in the identification. This species has not been observed in any subsequent surveys, and most observations of this species are well to the south, between Mexico and California, with the nearest observations in eBird off the coast of Oregon. There is one specimen from British Columbia from 1891 (Campbell et al. 1990), and a number of unconfirmed sightings in BC since then. Thus, combined with Guiguet's own uncertainty about the sighting, we do not want to put any weight on this potential record and have omitted it from Table 1.

#### A.5.8. Pink‐Footed Shearwater—*Ardenna creatopus*

Guiguet observed small numbers of individuals periodically starting in mid‐July, and he noted that this species seemed to arrive in the area later than others. Approximately 5–10 individuals were observed in the area on September 2, 2016 (H. Carolsfeld, personal communication).

#### A.5.9. Flesh‐Footed Shearwater—*Ardenna carneipes*

This species was observed regularly by Guiguet on the banks 29 km west of Yáláƛi and recorded as a “Pale‐footed Shearwater.” A flock of 50–60 birds was observed on June 13, 1948, with the numbers dwindling by mid‐August (Guiguet 1953). There have been no records of this species in the other surveys included in this study, and the nearest observations in eBird are from 40 to 50 km away.

#### A.5.10. Short‐Tailed Shearwater—*Ardenna tenuirostris*

Recorded by Guiguet as a “Slender‐billed Shearwater,” he noted that this species was uncommon in 1948. Guiguet collected one specimen on June 26, 26 km west of Goose Island. The bird had landed on “chum” behind the boat. As the specimen still had an apparent brood patch, Guiguet speculated that “this individual had only recently arrived from the breeding grounds below the equator” (Guiguet 1953). No other surveys have detected this species at Yáláƛi; however, it has been recorded in pelagic habitats on the Central Coast of BC on eBird.

#### A.5.11. Sooty Shearwater—*Ardenna grisea*

Guiguet noted that this was the most common bird observed in pelagic waters, and collected specimens in May with brood patches, and throughout the molting period. The molt was nearly completed on specimens collected on August 13, 1948. Sooty Shearwater was also recorded in CoastWatch surveys on July 6, 2010, where four individuals were noted in the air south of Gosling Rocks. On eBird, V. Bowersox reported one individual on Gull Island in 2011, and J. Reynolds reported 15 individuals in waters to the east of the islands. Additionally, 20–50 individuals were seen from Snipe Island in 2016 (H. Carolsfeld, personal communication).

#### A.5.12. Sabine's Gull—*Xema sabini*

Lone individuals of this species were observed three times by Guiguet, on June 9 and June 26 in pelagic waters, and on July 28, 1948, in the nearshore area. He noted that large flocks of small gulls observed at a distance in August may have been of this species. There are no other records of this species from Yáláƛi in subsequent surveys included in this study; however, there are records for the Central Coast of BC on eBird.

#### A.5.13. Black‐Legged Kittiwake—*Rissa tridactyla*

“Kittiwakes” appear in Guiguet's table of pelagic species (figure 19, Guiguet 1953), where he recorded between 40 and 60 individuals. We suspect this may have been an error, as there is no associated species description, whereas every other species detected does have such a description. There are also no field notes referring to kittiwakes. Under the Bonaparte's Gull (*Larus philadelphia*) description, Guiguet mentioned that there was a strong possibility of kittiwakes occurring, although none were recorded. See also below, under Bonaparte's Gull.

#### A.5.14. Bonaparte's Gull—*Chroicocephalus philadelphia*

Guiguet observed a single individual recorded in May 1948, which “was the only positive record for the island proper” (Guiguet 1953). However, he noted that some large flocks of gulls observed at a distance in August were “probably of this species.”. In the table of pelagic species (figure 19, Guiguet 1953), Guiguet recorded fewer than 10 Bonaparte's Gulls, and between 40 and 60 kittiwakes. We suspect that he might have decided to record these gulls as kittiwakes in the table after all. Although not detected by CoastWatch in 2010, the SFU research team in 2011, nor as part of the 100 Islands Project surveys in 2015, this species is commonly observed in the general area, with the closest observation on eBird being one by J. Reynolds near adjacent Hunter Island (~13 km away).

#### A.5.15. Red Phalarope—*Phalaropus fulicarius*

Guiguet recorded two individuals on July 28, and noted that at this time “fishermen on the banks reported large flocks of ‘Coho birds’ that may have been of this species”. He noted that the migration mostly passes much further west. There are no detections of this species by any contemporary surveys, nor in the 20 km grid square centered around Goose Island on eBird.

#### A.5.16. Red‐Necked Phalarope—*Phalaropus lobatus*

Guiguet observed this species as Northern Phalarope, migrating northward from May 22 to 27, 1948, and migrating southward starting June 26; “by July 13 they were extremely numerous throughout Queen's Sound feeding in the tide lines”. In mid to late July 2010, CoastWatch observed small flocks of this species just off North Beach, West Beach, and the Gosling Rocks (Figure 2). This species was also recorded by eBird observer H. Hall near Duck Island on July 18, 2017. H. Carolsfeld additionally observed between two and five individuals from Snipe Island on September 2, 2016 (H. Carolsfeld, personal communication).

### A.6. Swallows

#### A.6.1. Bank Swallow—*Riparia riparia*

Guiguet observed and collected a single individual flying over the lagoon on July 21, 1948. None have been observed at Yáláƛi since.

#### A.6.2. Violet‐Green Swallow—*Tachycineta thalassina*

Guiguet observed a single individual feeding over the lagoon on June 6, 1948. No others have been recorded since.

#### A.6.3. Barn Swallow—*Hirundo rustica*

Two Barn Swallows were observed by Guiguet feeding with the Violet‐green Swallow on June 6, 1948. Barn Swallows are common breeders on buildings in Wágļísļa (Bella Bella) and nearby Shearwater (Denny Island), approximately 30 km to the northeast, but despite the presence of two cabins on Goose Island, there have been no new records of this species on the islands since Guiguet's sightings.

### A.7. Warblers

#### A.7.1. Yellow Warbler—*Setophaga petechia*

Christine Rock of the 2011 SFU field crew saw one individual of this species on southern Goose Island on May 18, 2011. A singing individual was also observed on Duck Island on May 7, 2015, by the 100 Islands field crew.

#### A.7.2. Wilson's Warbler—*Cardellina pusilla*

Although not observed by Guiguet in 1948 or CoastWatch in 2010, this species was observed by our field teams in both 2011 and 2015. This species has records on eBird, with four observations by J. Reynolds on southern Yáláƛi and nearby Duck and Swan Islands in 2011, and four individual birds in total detected by J. Kennedy and the 100 Islands Project on Gosling Island in 2015.

#### A.7.3. Townsend's Warbler—*Setophaga townsendi*

Guiguet did not record any observations of this species in 1948. This is surprising as this species has been commonly observed in the more recent surveys by CoastWatch in 2010, Simon Fraser University researchers in 2011, by the 100 Islands Project team in 2015, as well as being frequently recorded on eBird.

#### A.7.4. Yellow‐Rumped Warbler—*Dendroica coronata*

On April 17, 2010, a male individual of the Myrtle subspecies, with an immaculate white throat, was detected by CoastWatch while singing on the south edge of Goose Island, where the conifers meet deciduous shrubs, near the Goose Island Anchorage. This species was not detected by C.J. Guiguet in 1948 nor in any other subsequent field surveys. There are also no records of this species in the area on eBird.

### A.8. Vagrants

#### A.8.1. House Sparrow—*Passer domesticus*

As recorded on eBird, on May 12, 2015, J. Reynolds and J. Kennedy were very surprised to encounter two individuals of this species near the northeastern tip of Duck Island.

#### A.8.2. Eurasian Collared Dove—*Streptopelia decaocto*

As part of the 100 Islands Project, we detected one individual of this species on June 18, 2015, on Gosling Island. B. Starzomski also recorded an individual on Snipe Island on May 11, 2015. J. Reynolds detected two individuals on the south end of Goose Island on May 25, 2011 and a single individual on May 27, 2011.

#### A.8.3. Northern Mockingbird—*Mimus polyglottos*

J. Reynolds and B. Starzomski share the only eBird record of this species on the islands, with a single individual feeding among beach logs on the northern tip of Goose Island on June 24, 2019.

### A.9. Uncommon Species

#### A.9.1. Northern Goshawk—*Accipiter gentilis*

This species was recorded only by Guiguet and reported in his published account as, “a large accipiter observed working over the muskegs on June 20, was believed to be of this species” (Guiguet 1953).

#### A.9.2. Western Screech‐Owl—*Megascops kennicottii kennicottii*

Guiguet observed at least two pairs of these owls nesting on the islands and collected the juveniles just after they left the nest on June 30, 1948. No Western Screech‐owls were recorded in any subsequent surveys of Yáláƛi, but they have been recorded on nearby islands on the Central Coast of BC, including Wágļísļa (Bella Bella) and Shearwater on Denny Island.

#### A.9.3. Western Tanager—*Piranga ludoviciana*

Guiguet recorded a pair on August 16, 1948, near the lagoon, comprising the only record for the islands.

#### A.9.4. Brown‐Headed Cowbird—*Molothrus ater*

On August 11, 1948, Guiguet observed and collected an immature female cowbird that had arrived on a beach with a flock of Semipalmated Plovers. Cowbirds have not been recorded on the islands in any subsequent surveys.

**TABLE A1:** Mammalian species present at Yáláƛi (the Goose Island Archipelago), Haíłzaqv territory, on the Central Coast of British Columbia, Canada, in two time periods.

English name (Haíłzaqvḷa name)	Latin name	1948	2010
Eastern Deer Mouse (H̓skáníqs)	*Peromyscus maniculatus*		
Long‐tailed Vole (H̓skáníqs)	*Microtus longicaudus*		
Dusky Shrew (H̓skáníqs)	*Sorex obscurus*		
Silver‐haired Bat (H̓ṃ̓?ázu)	*Lasionycteris noctivagans*		
Yuma myotis (H̓ṃ̓?ázu)	*Myotis yumanensis*		
Fin Whale	*Balaenoptera physalus*		
Humpback Whale (Ǧvi̓ṃ́)	*Megaptera novaeangliae*		
Dall's Porpoise	*Phocoenoides dalli*		
Steller Sea Lion (Máwák̓)	*Eunetopias jubata*		
Northern Fur Seal (Núkví)	*Callorhinus ursina*		
Harbor Seal (Sagvṃ́)	*Phoca vitulina*		
American Mink (Kvṇ̓á)	*Neogale vison*		
North American River Otter (K̓vḷá)	*Lontra canadensis*		
Gray Wolf (K̓vsḷs or K̓vḷsis)	*Canis lupus*		
Sitka Black‐tailed Deer (Qám̓ílá)	*Odocoileus hemionus sitkensis*		
North American Beaver (Qvúlúnṇ)	*Castor canadensis*	*	
Sea Otter (Q̓asá)	*Enhydra lutris*		

*Note:* Detections made by Charles J. Guiguet in the earlier time period (1948) are shaded dark red. Contemporary detections are in light red and come from CoastWatch surveys conducted in 2010. An asterisk (*) for North American beaver denotes that Guiguet did not detect live animals but noted signs of beaver activity. The gray cells in the 2010 column mark species which were not explicitly looked for during the CoastWatch surveys.
